# Banded karyotype of the Konya wild sheep (*Ovis orientalis anatolica* Valenciennes, 1856) from Turkey

**DOI:** 10.3897/compcytogen.v5i2.1151

**Published:** 2011-07-01

**Authors:** Atilla Arslan, Jan Zima

**Affiliations:** 1 Department of Biology, Faculty of Science, Selcuk University, TR-42031 Konya, Turkey; 2 Institute of Vertebrate Biology, Academy of Sciences of the Czech Republic, CZ-603 65 Brno, Czech Republic

**Keywords:** chromosomes, *Ovis*, sheep domestication, Turkey

## Abstract

Thekaryotype, C-banding, and nucleoar organizer regions (NORs) of eight specimens ofKonya wild sheepfrom Turkey were examined. The complement included six large metacentric autosomes, 46 acrocentric autosomes of decreasing size, a medium-sized acrocentric X chromosome, and a small bi-armed Y chromosome (the diploid chromosome number 2n=54, the number of autosomal arms NFa=58, the number of chromosome arms NF=61). G-banding allowed reliable identification of all the chromosome pairs and the pairing of homologous elements. All the autosomes possessed distinct centromeric or pericentromeric C-positive bands. The X chromosome had a pericentromeric C-positive band, and the Y chromosome was entirely C-heterochromatic. The NORs were located in the terminal regions of the long arms of three metacentric and two acrocentric autosomes. The karyotype of the Konya wild sheep and its banding patterns are quite similar to chromosome complement reported in domestic sheep and European mouflon.

## Introduction

The systematics of the genus *Ovis* (Linnaeus, 1758) is rather complicated and there are different opinions in respect of the number of species and the actual species borders (Nadler et al. 1973a, b, Shackleton et al. 1997, Grubb 2005). One of the main problems is the origin and taxonomic position of the domestic sheep. For the domestic sheep a large number of possible ancestral species and subspecies exist, and several Eurasian wild sheep taxa have been proposed as ancestors of domestic sheep or are believed to have contributed to specific breeds. Mouflons in the Mediterranean islands [*Ovis musimon* (Pallas, 1811); *Ovis ophion* Blyth, 1841] introduced to various parts of Europe are generally evaluated as feral populations of ancient domestic stocks (e.g. Hiendleder et al. 1998a, b). Phylogenetic analyses on mtDNA restriction fragment length polymorphism of Eurasian breeds of domestic sheep demonstrated two maternal origins among modern sheep breeds and indicated wild ancestors different from the previously considered urial (*Ovis vignei* Blyth, 1841) and argali sheep [*Ovis ammon* (Linnaeus, 1758)] (Hiendleder et al. 1998b). In a subsequent study, Hiendleder et al. (2002) confirmed two well-separated mtDNA lineages (European and Asian) suggesting domestication from two distinct wild populations.

The domestic sheep and its wild ancestors are sometimes considered conspecific, in which case the name *Ovis orientalis* has generally been used, although some authors use the name *Ovis aries* for both wild species and its domestic descendants (Grubb 2005). Likely candidates for wild ancestors of the European lineage of domestic sheep are populations found in Turkey and western Iran. At the species or subspecies level, these populations are currently referred to *Ovis orientalis*, *Ovis orientalis* Gmelin, 1774,*Ovis gmelinii* Blyth, 1841, *Ovis anatolica* Valenciennes, 1856, *Ovis ophion* or *Ovis musimon* (Kaya et al. 2000, 2004, Kryštufek and Vohralík 2009). The Konya wild sheep is a westernmost taxon of extant wild sheep endemic to Central Anatolia in Turkey, and it is usually referred to *Ovis orientalis anatolica* (Kryštufek & Vohralík, 2009) or *Ovis gmelinii anatolica* (Kaya et al. 2000). The only population is currently living in the area east of Konya (Bozdağ Protection of Wildlife). The Konya wild sheep was widespread 50 years ago, but the numbers have decreased rapidly because of harsh weather conditions, predator pressure, and hunting. The most severe population decline appeared in 1960s and 1970s when the population size of 30–40 individuals was reported (Kaya 1991, Kaya et al. 2000, Sezen et al. 2004). The abundance has later increased owing to protection, and the current numbers are estimated at several hundreds of heads (600 individuals reported in 2006; Yalçın et al. 2010). The apparently last surviving population inhabits a large protected area surrounded with a fence providing open steppe habitats in altitude ranging between 1000–1735 m above sea level. The Konya wild sheep is not phenotypically akin to the European or Mediterranean mouflons, because it displays the type of horn configuration typical of Asiatic wild sheep (Yalçın et al. 2010).

Chromosome research has contributed significantly to understanding of the evolutionary divergence among sheep taxa, and different chromosome numbers were found in individual geographic populations. The European mouflon and wild sheep from western Palaearctic (*Ovis orientalis*) have the same diploid number of 54 chromosomes as the domestic sheep. In the Iranian populations of urial sheep the diploid number of 58 chromosomes was recorded, and hybrid zones with polymorphic karyotype were found in the areas of contact with *Ovis orientalis* (Nadler et al. 1973a, Valdez et al. 1978). The argali sheep possess 56 chromosomes in their complements, 2n=52 was found in *Ovis nivicola* Eschscholtz, 1929, and 2n=54 in both the American species (Vorontsov et al. 1972a, b, Nadler et al. 1973a, b, Korobitsyna et al. 1974, Bunch et al. 1976, 1998, 2000, Lyapunova et al. 1997). The chromosomal findings support the view that wild sheep of the urial or argali types did not contribute to domestication of the sheep. However, despite the differences in chromosome number, different species of the genus can hybridize in captivity (Vorontsov et al. 1972b, Nadler et al. 1973b).

The karyotype of the Konya wild sheep was studied by Kaya et al. (2004), and the diploid number of 54 chromosomes was ascertained. Only a description of the basic karyotype characteristics was given, without any illustrations. Subsequently, Kırıkçi et al. (2003) reported the G-banding pattern of chromosomes of this population. In the present paper, we provide detailed description of the karyotype of the Konya wild sheep, a possible ancestor of domestic sheep, using various chromosome banding techniques.

## Material and methods

**Figure 1.**
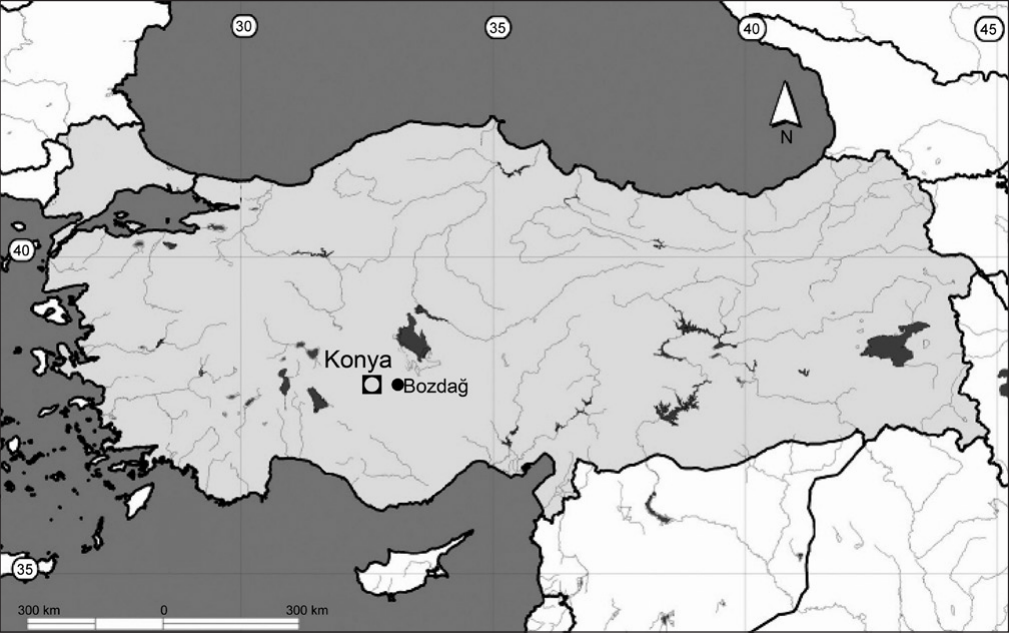
Collecting locality in the province of Konya, Anatolia, Turkey (●).

We examined eight specimens of Konya wild sheep including four females and four males. All the studied animals originated from Bozdağ in the Konya province, Turkey (Fig. 1). Chromosomal preparations were made using peripheral blood culture for 72 hr at 37oC in medium supplemented with 15% fetal calf serum and 0.024 % phytohaemagglutinin (Freshney 1990). The chromosome slides were conventionally stained with Giemsa. G-banding by trypsin treatment stained with Giemsa (GTG) was performed (Seabright 1971). Constitutive heterochromatin and nucleolus organizer regions (NORs) were detected with C-banding (Sumner 1972) and Ag-NOR staining (Howell and Black 1980), respectively.

## Results and discussion

**Figure 2.**
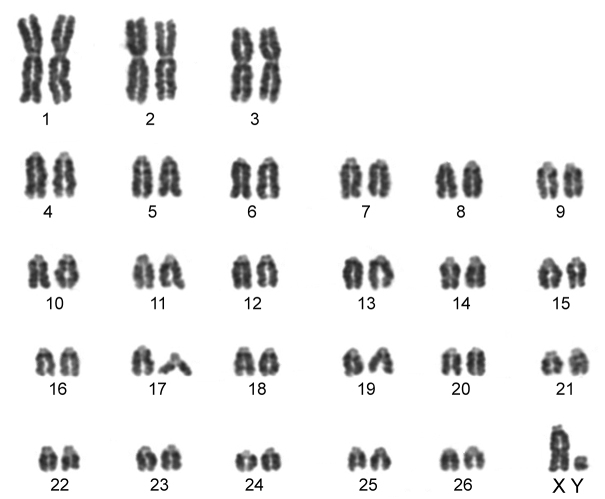
Conventionally stained karyotype of the Konya wild sheep with 2n=54. There are three pairs of large metacentric autosomes and 23 pairs of acrocentric autosomes. The X chromosome is acrocentric and the Y chromosome metacentric.

**Figure 3.**
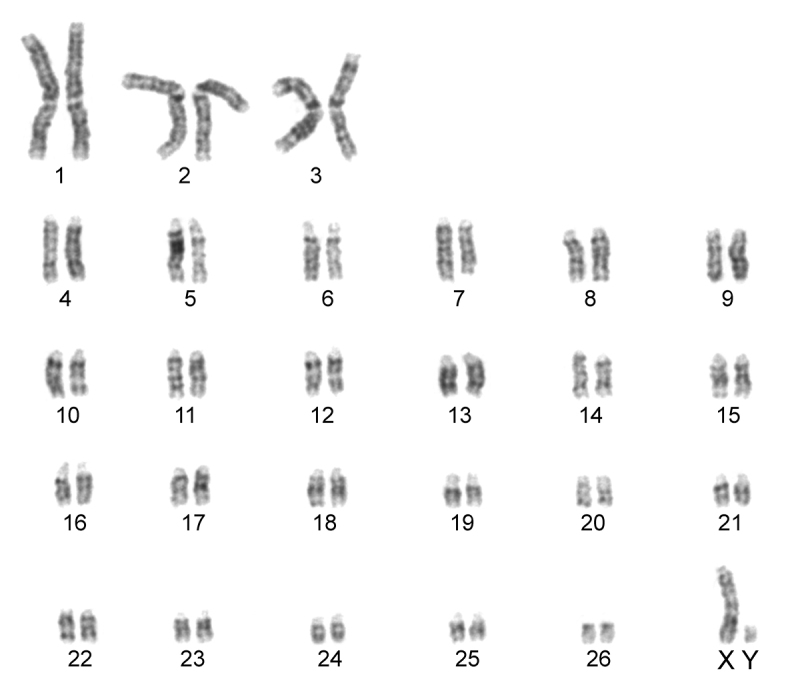
G-banded karyotype of the Konya wild sheep. All the chromosomes in the complement can be identified and differentiated according to their G-banding pattern.

**Figure 4.**
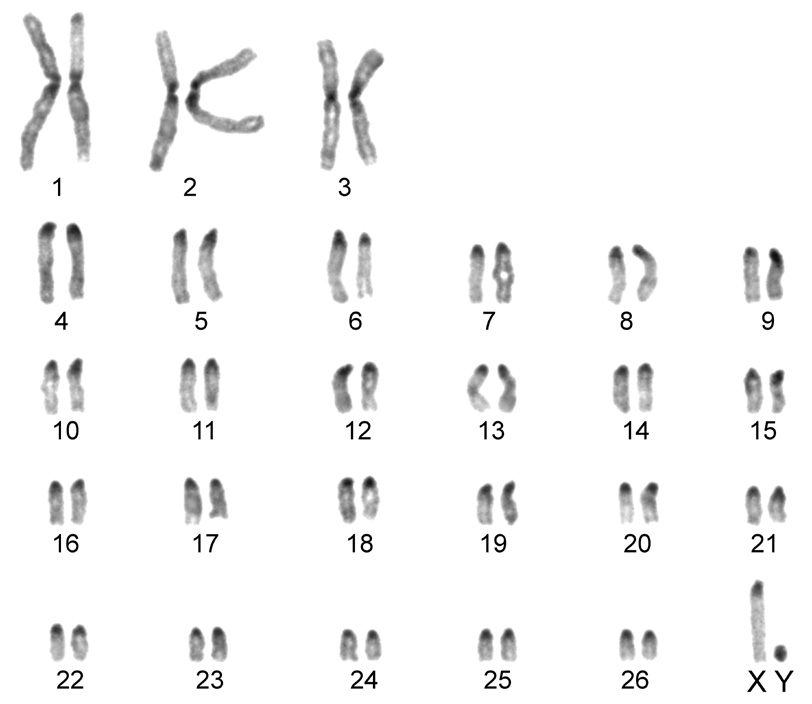
C-banded karyotype of the Konya wild sheep. Distinct pericentromeric dark bands are apparent in all the autosomes and the X chromosome. The Y chromosome is stained entirely positively.

**Figure 5.**
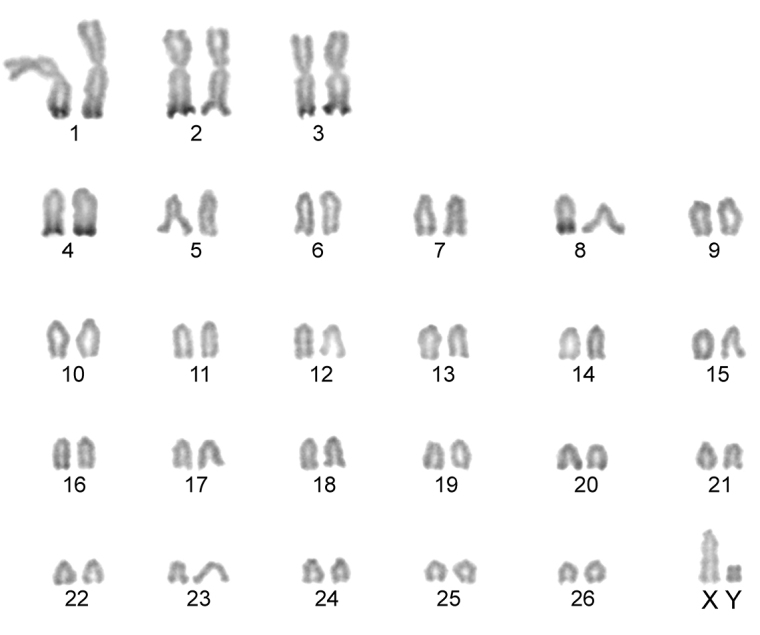
Silver-stained karyotype and the distribution of NORs in the Konya wild sheep. The NORs are localized in telomeric areas of the long arms of the three metacentric autosomal pairs (nos. 1, 2, 3) and two acrocentric autosomal pairs (nos. 4, 8). The heterozygous condition of the NORs in the acrocentric pair no. 8 was found consistently in various cells and different examined individuals.

The karyotype contained 54 chromosomes (Fig. 2) including three large metacentric autosomal pairs (no. 1–3) and 23 acrocentric autosomal pairs of decreasing size (nos. 4–26; NFa=58). Tiny short arms were observed in most of the acrocentric autosomes. The X chromosome was a large acrocentric with a distinct short arm, whereas the Y chromosome was metacentric and the smallest element of the complement (NF=61). All the autosomes and both the sex chromosomes were reliably identified on the basis of their unique G-banding patterns (Fig. 3). The C-banded karyotype of Konya wild sheep is illustrated in Fig. 4. All the autosomes possessed distinct and large centromeric C-positive bands which usually extended in the neighbouring pericentromeric areas. The X chromosome had a pericentromeric C-positive band, and the Y chromosome was entirely heterochromatic. Silver-nitrate staining revealed that NORs were localized in the distal position on the long arms of three large metacentric (no. 1–3) and two acrocentric (nos. 4 and 8) autosomes (Fig. 5). All the NORs were observed in both the homologous chromosomes, except those of the pair no. 8 in which a heterozygous condition with the signal present in only one homologue was recorded in four specimens.

The obtained karyotypic data confirm possible close relationships between the Konya wild sheep and domestic sheep. The diploid number of chromosomes and their morphology expressed in the number of chromosomal arms and the proportion of the metacentric and acrocentric autosomes are the same in both complements. The detailed structure of banded chromosomes of the Konya wild sheep appears quite similar in comparison with the standard chromosomal complement of the domestic sheep (cf. Ansari et al. 1999; ISCNDB 2000). The G-banding pattern observed in individual autosomes and the sex chromosomes is apparently identical between the karyotype of the Konya wild sheep and domestic sheep. The amount of pericentromeric C-heterochromatin is noticeably large in both the complements, and the Ag-NOR sites are localized in the same homologous chromosomes. Heteromorphism in the number of active NORs found in the acrocentric autosomal pair no. 8 may have resulted from the small sample size used in this study.

A considerable degree of similarity can also be demonstrated by comparison of our results with previously reported differentially stained chromosomes of various wild sheep taxa (Nadler et al. 1973b, Valdez et al. 1978, Bunch et al. 1998, 2000, Kırıkçi et al. 2003). Banding pattern in chromosomes and the distribution of NORs are also quite similar to that reported for European mouflon (Slavíčková et al. 1985). We may conclude that the chromosomal data support the possible position of the Konya wild sheep as one of the ancestors of domestic sheep. The surviving population should be subjected to further extensive genetical research, and its strict protection should be maintained.
